# IADD: An integrated Arabic dialect identification dataset

**DOI:** 10.1016/j.dib.2021.107777

**Published:** 2021-12-30

**Authors:** Jihad Zahir

**Affiliations:** LISI laboratory, Cadi Ayyad University, Marrakesh, Morocco

**Keywords:** Dialect identification, Arabic language, Geographic disaggregation, Web mining

## Abstract

Arabic language has different variants that can be roughly categorized into three main categories: Classical Arabic (CA), Modern Standard Arabic (MSA) and Dialectal Arabic (DA). There are subtle differences between MSA and CA in terms of syntax, terminology and pronunciation. However, Dialectal Arabic (DA) significantly differs from CA and MSA in that it reflects geographic location of the speaker, or at least the country of origin, if mobility factors are taken into account. This paper presents IADD, an Integrated dataset for Arabic dialect identification, that contains 135,804 texts representing Arabic dialects from 5 regions and 9 countries. IADD dataset is created, from the combination of subsets of five corpora, to support the task of automatic Arabic dialects detection.

**Specifications Table**

**Table:** 

Subject	Data Science
Specific subject area	The dataset relates to automatic dialect identification which is a natural language processing task that focuses on automatically detecting the dialect in which a text is written.
Type of data	A JSON file with 135,804 elements with four (key, value) each.
How data were acquired	The dataset is created by combining subsets of 5 corpora
Data format	Filtered
Data collection	Integrated Arabic Dialect iDentification Dataset (IADD) is created in two steps: 1) Data sources identification and 2) data preparation and insertion. Five publicly available corpora were identified, analyzed and filtered to build IADD as described in section 2. Each corpus supports a set of multiple dialects including Levantine, Tunisian, Egyptian, Maghreb, Iraqi and Gulf dialects. Different text types, such as tweets and Facebook comments, are supported. Corpora that were considered to build IADD were published between 2011 and 2018.
Data source location	The list of the primary data sources used to create IADD dataset is as follows. Primary data sources: AOC [1]: https://github.com/sjeblee/AOC DART [2]: https://www.dropbox.com/s/jslg6fzxeu47flu/DART.zip?dl=0 PADIC [3]:https://sourceforge.net/projects/padic/ SHAMI [4]: https://github.com/GU-CLASP/shami-corpus/tree/master/Data TSAC [5]:https://github.com/fbougares/TSAC
Data accessibility	Data is hosted on a public repository Repository name: GitHub Direct URL to data: https://github.com/JihadZa/IADD

## Value of the Data

• The proposed dataset not only covers different Arabic dialects but also different types of texts that are typically found on the web. IADD contains examples of tweets, Facebook posts and online comments, this aspect is important to generate classifiers that handle different types of textual content.
• The dataset can benefit the natural language processing community as it can be used to build and compare classifiers that automatically predict the dialect expressed by a text written in Arabic. Digital data analysts can also use the dataset to infer the geographic origin of Arabic-speaking web users by identifying the dialect they use in their interactions online.
• IADD might be also used to support corpus-based dialectometry and study geo-linguistic variations between Arabic dialects.
• The proposed dataset can be valuable in at least two domains:
  • Marketing Analytics: The accurate identification of the demographic characteristics is crucial in audience analysis, this dataset represents a resource to support the automatic identification of the geographic origin of reviews and comments authors.
  • Public opinion disaggregation: Opinion mining (i.e. sentiment analysis) has been extensively used as a tool to gauge public opinion toward a given subject. Classical approaches are limited to polarity and objectivity analysis. With the proposed resource, opinions can be disaggregated by geographic location providing in-depth insight into public opinion and uncovering potential disparities within the community of Arabic-speaking web users.

## 1 Data Description

The objective was to build a diverse and large dataset with a wide coverage of dialects and types of textual content, which ensures a better generalization of classification models. Integrated Arabic Dialect Dataset (IADD) is created in two steps: (1) Data sources identification and 2) data preparation and insertion. At the end of the process, IADD is stored in a JSON-like format with the following keys:

• *Sentence*: contains the sentence/ text;
• *Region*: stores the corresponding dialectal region (MGH, LEV, EGY, IRQ, GLF or general);
• *Country*: specifies the corresponding country, if available (MAR, TUN, DZ, EGY, IRQ, SYR, JOR, PSE, LBN);
• *DataSource*: indicates the source of the data (PADIC, DART, AOC, SHAMI or TSAC).

**Fig. 1:**
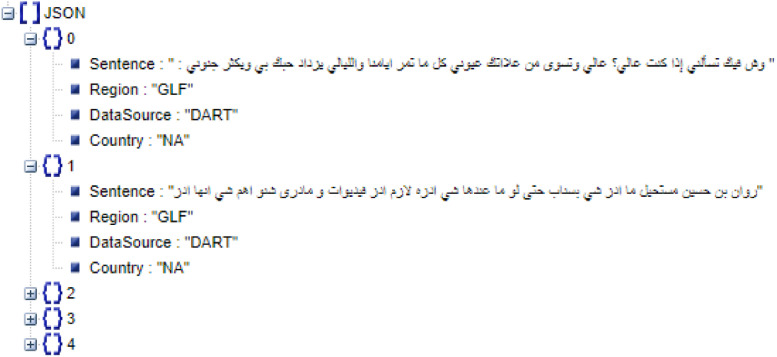
A record from IADD.

Fig. 1 presents examples of records from IADD.

**Fig. 2:**
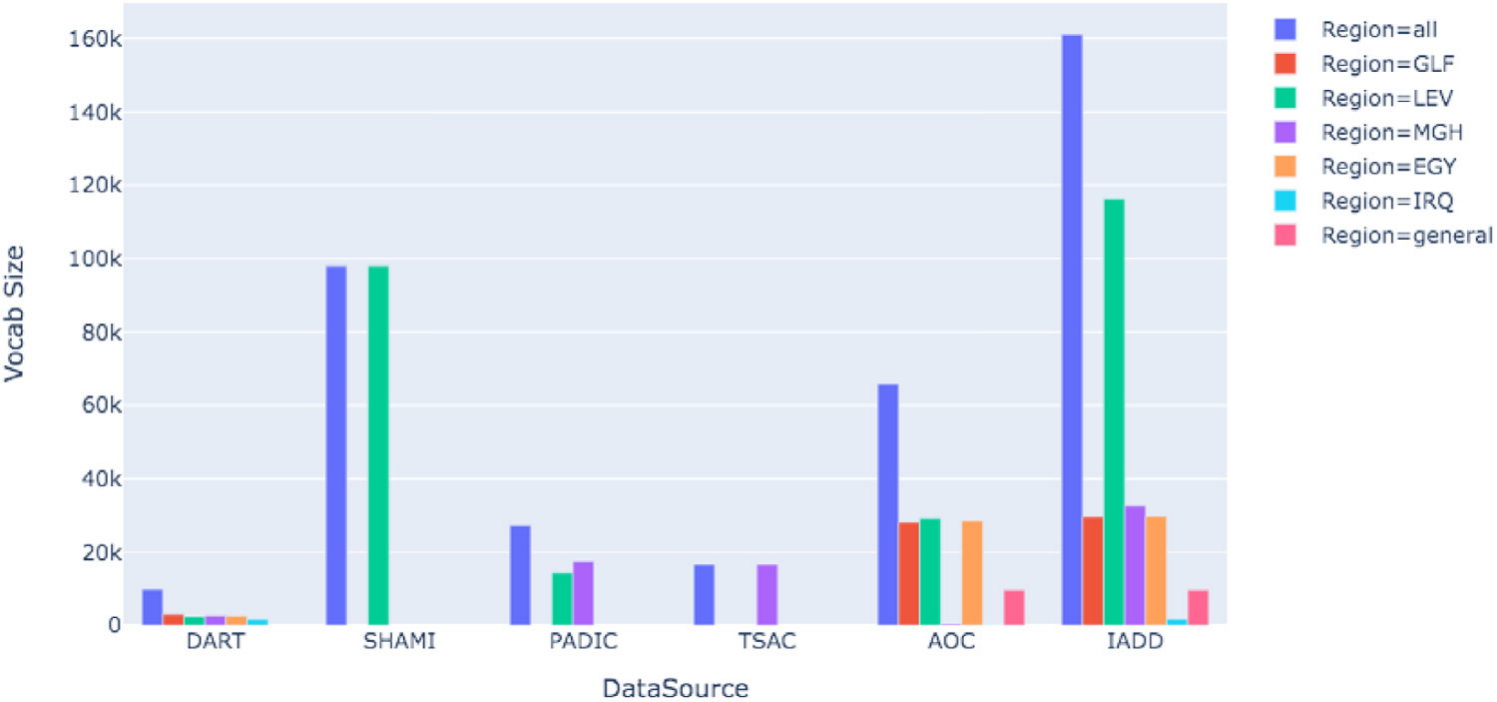
Vocabulary size by region and by data source.

Table 1 and Fig. 2 provide an overview of IADD, describing the number and percentage of sentences by region and country, and the vocabulary size. Average word count, characters count and number of stop words per sentence, for each regional dialect, are presented in Table 2.

**Table 1:** Detailed overview of IADD.

Region	Country	Sentences #

Maghrebi (MGH)	Algeria	14,426
	Morocco	7213
	Tunisia	11,998

**Total**		**33,996** (25%)

Levantine (LEV)	Palestine	17,855
	Jordan	7017
	Syria	44,972
	Lebanon	10,829

**Total**		**87,573** (≈64%)

Egypt (EGY)	Egypt	4837(3.6%)
Iraq (IRQ)	Iraq	216 (<1%)
Gulf (GLF)	−−−	6682 (≈5%)
general	−−−	2500 (≈2%)

**Total**		135,804 (100%)

**Table 2:** Data description.

Region	AVG word count	AVG # characters per word	AVG stop words #
Gulf (GLF)	15.69	4.49	2.41
Levantine (LEV)	13	4.05	2.13
Maghrebi(MGH)	6.71	3.38	1.15
Egypt (EGY)	23.78	4.38	3.89
Iraq (IRQ)	12.75	4.11	1.57

**Fig. 3:**
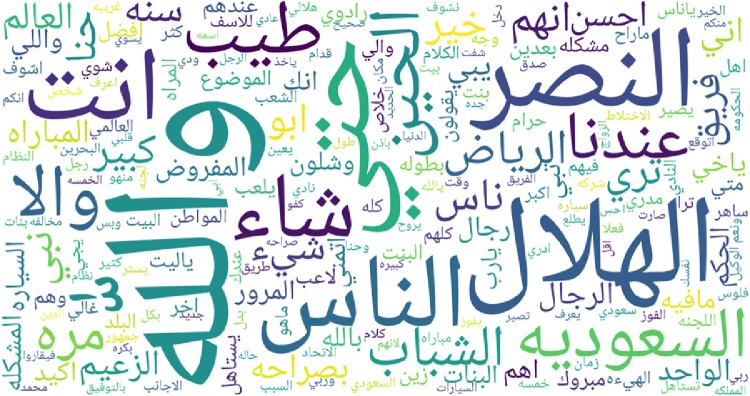
Most frequent words in Gulf dialect.

**Fig. 4:**
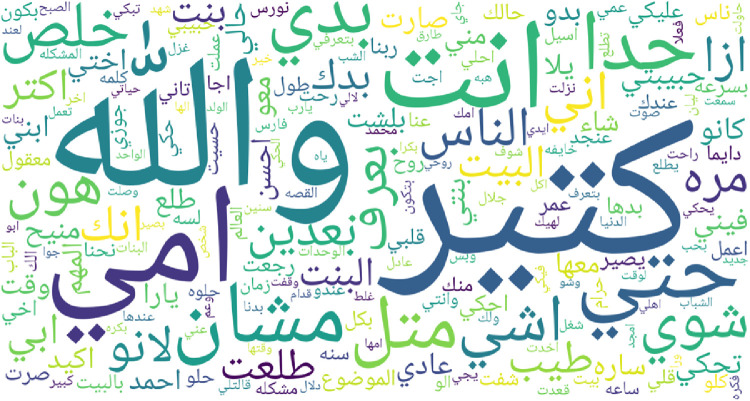
Most frequent words in Levantine dialect.

**Fig. 5:**
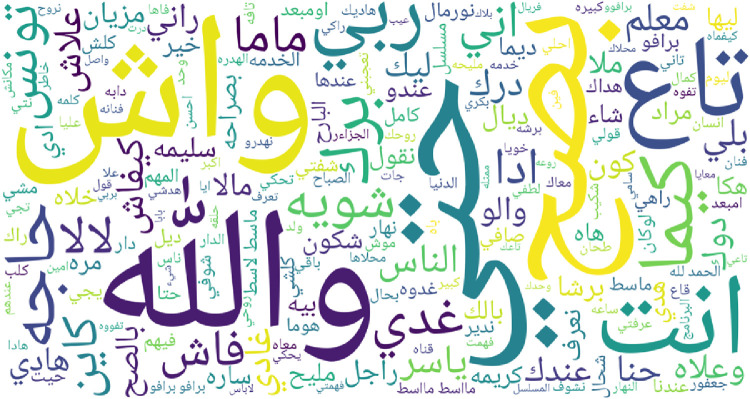
Most frequent words in Maghrebi dialect.

**Fig. 6:**
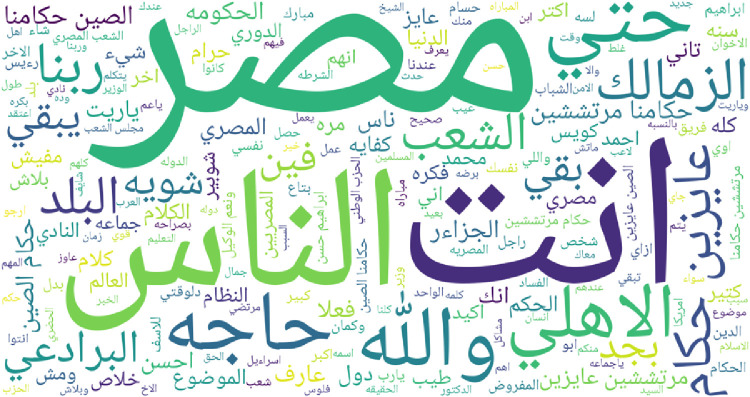
Most frequent words in Egyptian dialect.

**Fig. 7:**
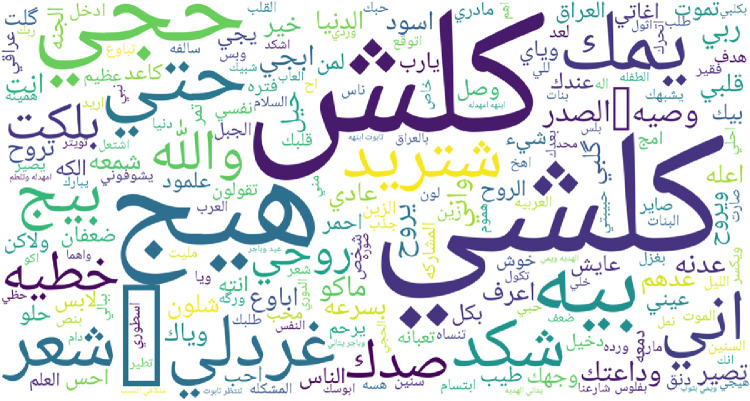
Most frequent words in Iraqi.

**Fig. 8:**
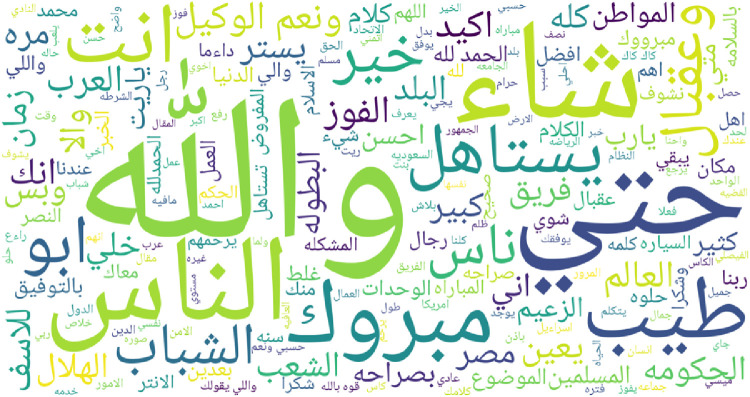
Most frequent words in language classified as ’general’.

To give an overview of most frequent words for each regional dialect supported by IADD, word clouds featuring top 200 words are presented in Fig. 3, Fig. 4, Fig. 5, Fig. 6, Fig. 7 and 8. Before plotting word clouds, a number of preprocessing steps have been conducted:

1. Letters normalization,
2. Digits and punctuation removal,
3. Latin characters removal,
4. Elongation removal,
5. Diacritics removal,
6. Stop words from modern standard Arabic were also removed while stop words that are dialect specific remained unchanged.

**Fig. 9:**
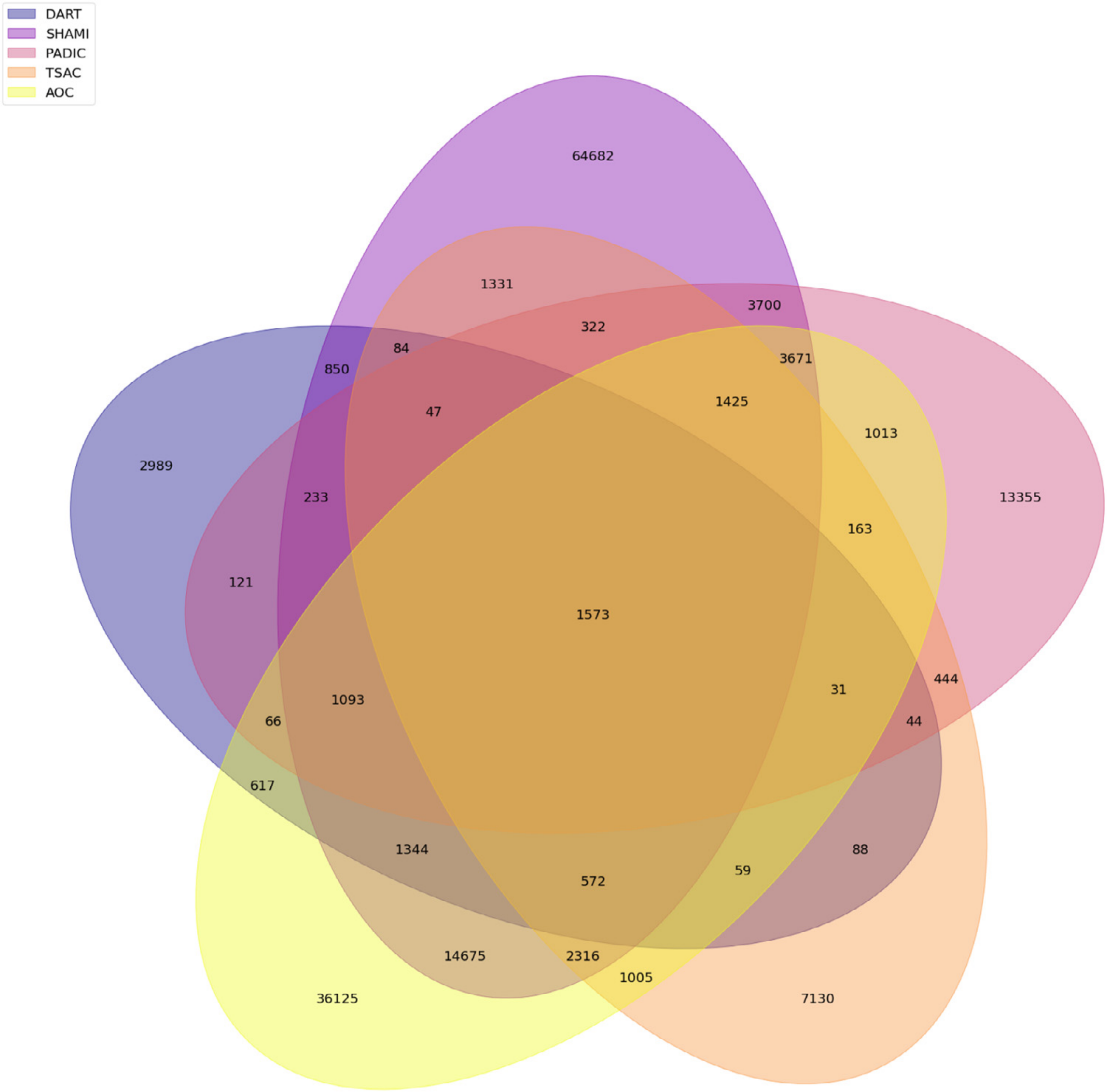
Common words between data sources.

**Fig. 10:**
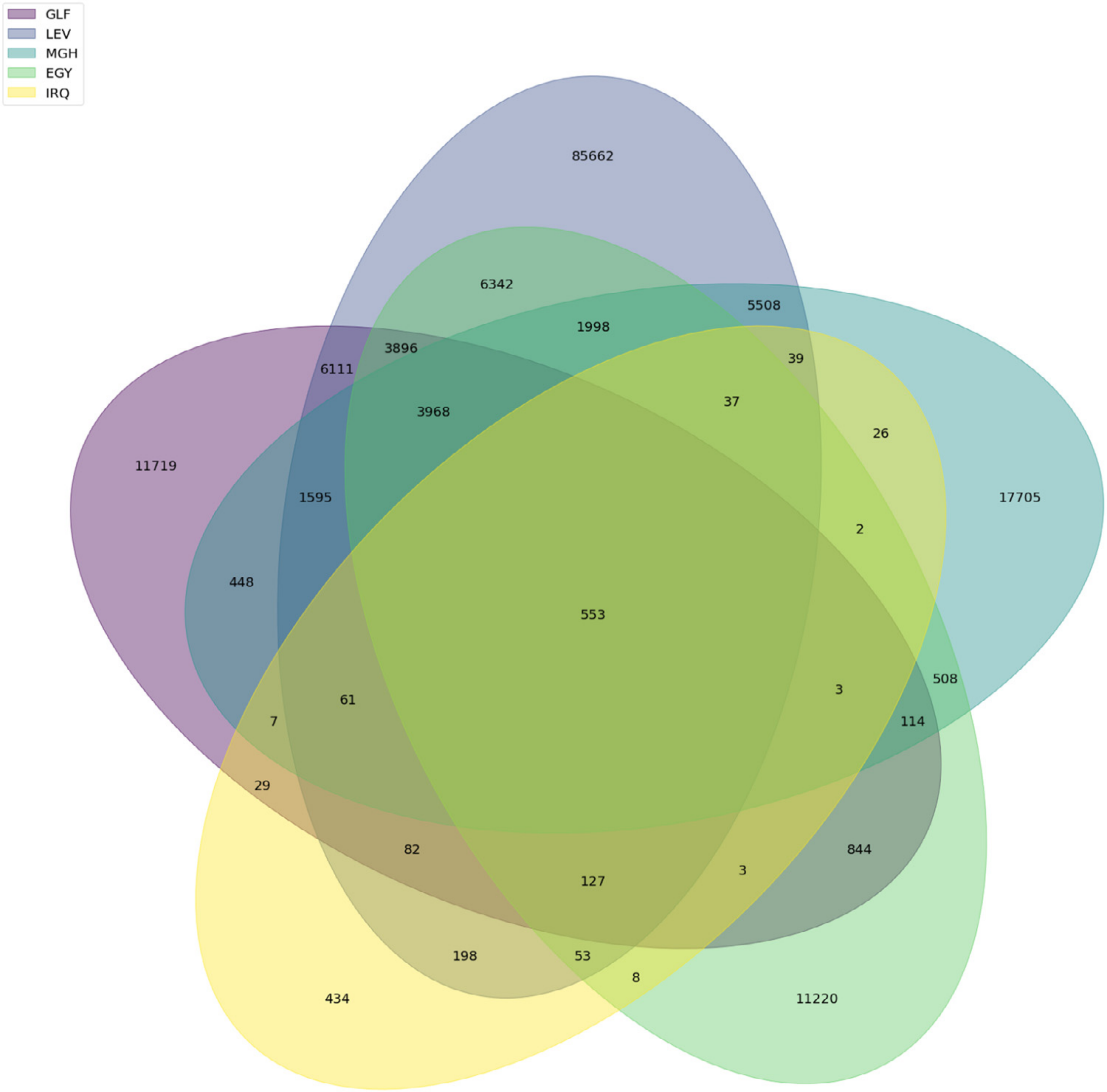
Common words between data sources.

Venn diagrams, presented in Figs. 9 and 10, show the numbers of common words between dialects’ vocabularies (Fig. 10) and between data sources’ vocabularies (Fig. 9). Figures show that there are 553 common words between dialects’ vocabularies while data sources’ vocabularies share 1573 words.

## 2 Experimental Design, Materials and Methods

### 2.1 Data sources identification

IADD is created from the combination of subsets of five corpora: DART, SHAMI, TSAC, PADIC and AOC. Each corpus supports a different set of dialects, as shown in Table 3. The Dialectal ARabic Tweets dataset (DART) [2] has about 25,000 tweets that are annotated via crowdsourcing while the SHAMI dataset [4] consists of 117,805 sentences and covers levantine dialects spoken in Palestine, Jordan, Lebanon and Syria. TSAC [5] is a Tunisian dialect corpus of 17,000 comments collected mainly from Tunisian Facebook pages. Parallel Arabic Dialect Corpus (PADIC) [3] is made of sentences transcribed from recordings or translated from MSA. Finally, the Arabic Online Commentary (AOC) dataset [1] is based on reader commentary from the online versions of three Arabic newspapers, and it consists of 1.4M comments.

**Table 3:** Description of corpora composing IADD.

		Supported Dialects	
Corpus	Source	Regional Level	Country Level
DART [2]	Twitter	Egyptian, Maghrebi, Levantine, Gulf, and Iraqi.	Egypt, Iraq.
SHAMI [4]	Twitter	Levantine.	Palestine, Jordan, Lebanon and Syria.
TSAC [5]	Facebook userscomments	Maghrebi.	Tunisia.
PADIC [3]	Manual transcription from recordings of conversations, movies or shows	Levantine and Maghrebi.	Syria, Palestine, Algeria, Morocco.
AOC [1]	Readers’ commentsin websites of Arabicnewspapers	Egyptian, Maghrebi, Levantine, Gulf, and Iraqi.	Egypt, Iraq.

### 2.2 Data preparation and insertion

Data preparation and insertion procedures, from each data source into IADD, are detailed below.

#### 2.2.1 SHAMI and TSAC

Sentences from SHAMI and TSAC are directly inserted in IADD. *Region* is set to “LEV” for SHAMI data and to “MGH” for TSAC data.

#### 2.2.2 DART

Regarding DART, besides the five groups of regional dialects (EGY, IRQ, GLF, LEV, MGH), it contains also an additional group named “Other”. The items corresponding to the “Other” category are discarded and are therefore not added in IADD.

#### 2.2.3 PADIC

Sentences in PADIC are initially classified into 6 categories of dialects: *ALGIERS, ANNABA, MODERN-STANDARD-ARABIC, SYRIAN, PALESTINIAN* and *MOROCCAN*.

• *ALGIERS* and *ANNABA* are two cities in Algeria. These tags are used to distinguish sentences written in Annaba dialect from those written in Algiers dialect.
• *MODERN-STANDARD-ARABIC* tag is associated to sentences written in MSA;
• *SYRIAN, PALESTINIAN* and *MOROCCAN* are dialects corresponding to Syria, Palestine and Morocco, respectively.

**Fig. 11:**
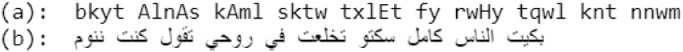
(a) Original Buckwalter encoded sentence from PADIC, (b) transliterated version to Arabic.

*ALGIERS, ANNABA* and *MOROCCAN* represent dialects from Maghrebi region. Therefore, all sentences annotated as such are mapped to region value “MGH”. Similarly, “LEV” is assigned to *SYRIAN* and *PALESTINIAN* sentences. At last, sentences holding the *MODERN-STANDARD-ARABIC* tag are discarded. Aside from that, as PADIC is publicly available in the format of an XML file that contains Buckwalter^1^ encoded sentence, every sentence is mapped to its Arabic version, before including it to IADD. Fig. 11 shows an example of a sentence before and after transformation.

#### 2.2.4 AOC

**Fig. 12:**
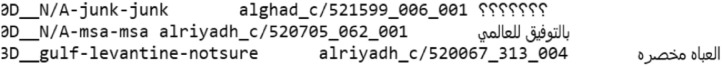
Example of discarded texts.

**Fig. 13:**
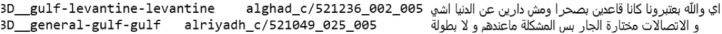
Example of texts included in IADD.

Texts in AOC dataset have 3 annotations given by 3 different reviewers. Annotators judged each text and assigned, according to their judgment, one of the following labels: “*notsure*”, “*junk*”, “*levantine*”, “*egyptian*”, “*gulf*”, “*’iraqi’*”, “*maghrebi*”, “*general*” and “*’msa’*”. Only texts with at least two identical annotations are considered. From these, texts annotated as “*msa*”, “*’junk’*” or “*notsure*” are discarded, as sentences with the “*msa*” tag are in modern standard language and the two other tags are associated with noisy and ambiguous sentences, respectively. Fig. 12 presents an example of discarded texts and in Fig. 13 is an example of texts that are kept and included in IADD.

## Declaration of Competing Interest

The author declares that there is no known competing financial interests or personal relationships which have, or could be perceived to have, influenced the work reported in this article.

## Footnotes

1 Buckwalter Transliteration is a reversible transliteration scheme used to write Arabic characters using Latin ASCII characters.
